# Supplementary figures and images for: Capturing variation in floral shape: a virtual3D based morphospace for Pelargonium
Source: PeerJ. 2020 Apr 2;8:e8823. doi: 10.7717/peerj.8823 (PMC7130111; doi:10.7717/peerj.8823)

A

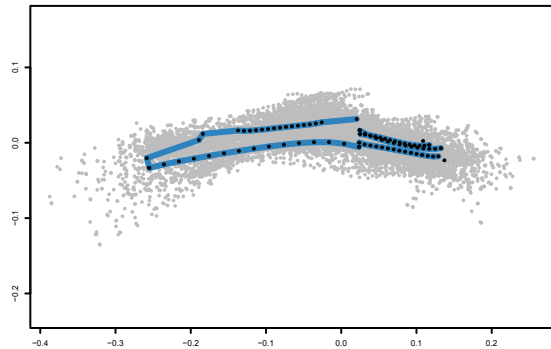

B

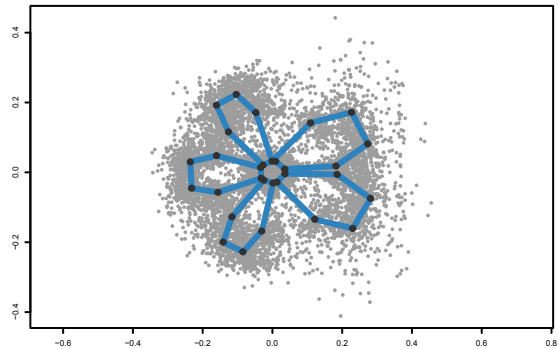

Supplement: Supplemental Information 5 — Supplementary figure showing mean shape and spread of Procrustes residuals per landmark for (A) TUBE and (B) PETAL datasets [file peerj-08-8823-s005.pdf]

PC scores TUBE dataset

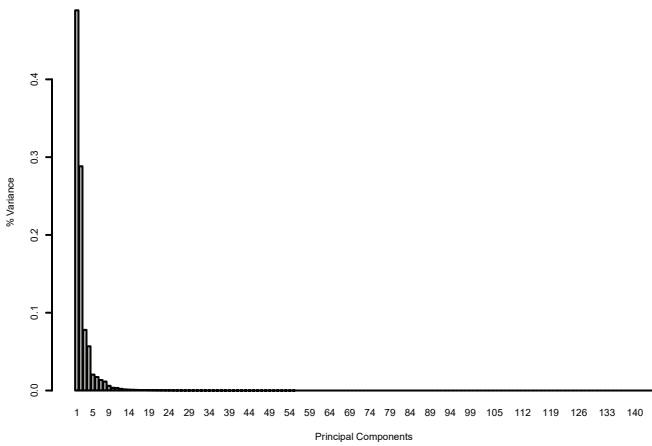

PC scores PETAL dataset

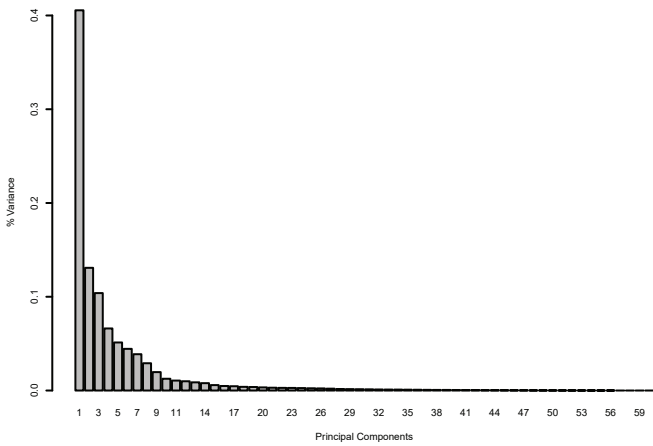

PC scores VIRTUAL3D dataset

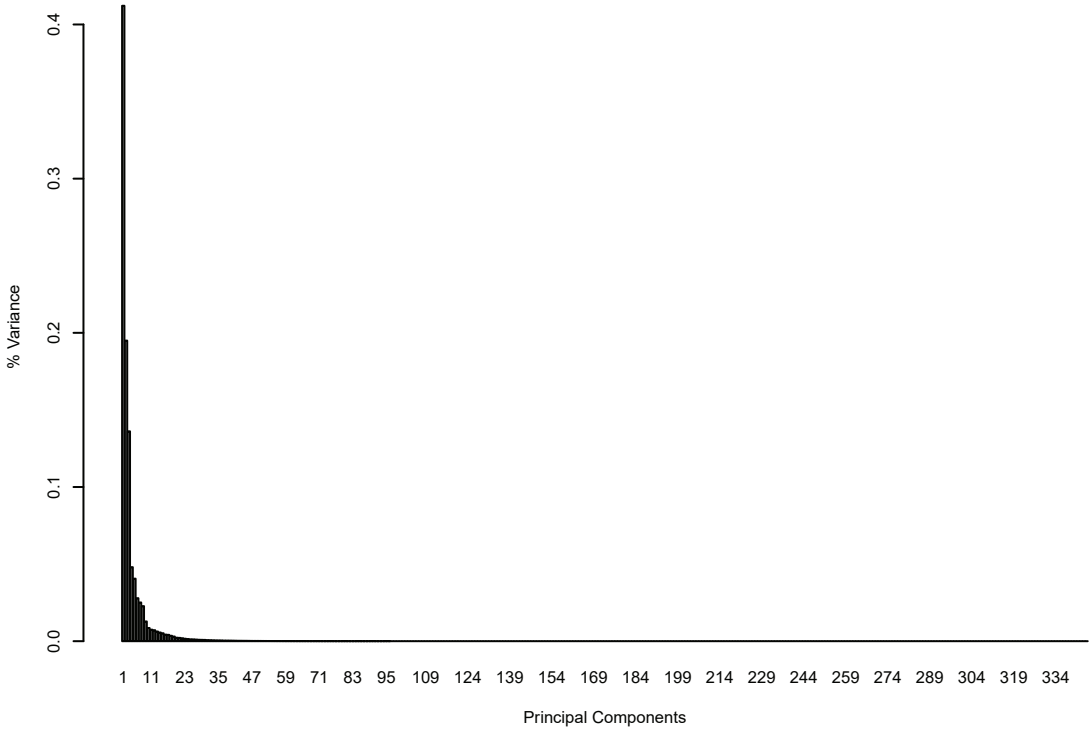

Supplement: Supplemental Information 6 [file peerj-08-8823-s006.pdf]
